# Inhibition of Slc39a14/Slc39a8 reduce vascular calcification via alleviating iron overload induced ferroptosis in vascular smooth muscle cells

**DOI:** 10.1186/s12933-024-02224-z

**Published:** 2024-05-29

**Authors:** Yierpani Aierken, Huqiang He, Runwen Li, Zipeng Lin, Tongjie Xu, Li Zhang, Ya Wu, Yong Liu

**Affiliations:** 1 Department of Vascular Surgery, The Affiliated Hospital, Southwest Medical University, No. 25, Taiping Street, Luzhou, 646000 Sichuan China; 2 Key Laboratory of Medical Electrophysiology, Ministry of Education & Medical Electrophysiological Key Laboratory of Sichuan Province, (Collaborative Innovation Center for Prevention of Cardiovascular Diseases) Institute of Cardiovascular Research, Southwest Medical University, Luzhou, 646000 China; 3 Department of General Surgery, The Affiliated Hospital, Southwest Medical University, Luzhou, 646000 China; 4 Metabolic Vascular Disease Key Laboratory of Sichuan Province, The Affiliated Hospital, Southwest Medical University, Luzhou, 646000 China

**Keywords:** Vascular calcification, Ferroptosis, Iron overload, Oxidative stress, Iron transport

## Abstract

**Background:**

Vascular calcification (VC) is an independent risk factor for cardiovascular diseases. Recently, ferroptosis has been recognised as a novel therapeutic target for cardiovascular diseases. Although an association between ferroptosis and vascular calcification has been reported, the role and mechanism of iron overload in vascular calcification are still poorly understood. Specifically, further in-depth research is required on whether metalloproteins SLC39a14 and SLC39a8 are involved in ferroptosis induced by iron overload.

**Methods:**

R language was employed for the differential analysis of the dataset, revealing the correlation between ferroptosis and calcification. The experimental approaches encompassed both in vitro and in vivo studies, incorporating the use of iron chelators and models of iron overload. Additionally, gain- and loss-of-function experiments were conducted to investigate iron’s effects on vascular calcification comprehensively. Electron microscopy, immunofluorescence, western blotting, and real-time polymerase chain reaction were used to elucidate how Slc39a14 and Slc39a8 mediate iron overload and promote calcification.

**Results:**

Ferroptosis was observed in conjunction with vascular calcification (VC); the association was consistently confirmed by in vitro and in vivo studies. Our results showed a positive correlation between iron overload in VSMCs and calcification. Iron chelators are effective in reversing VC and iron overload exacerbates this process. The expression levels of the metal transport proteins Slc39a14 and Slc39a8 were significantly upregulated during calcification; the inhibition of their expression alleviated VC. Conversely, Slc39a14 overexpression exacerbates calcification and promotes intracellular iron accumulation in VSMCs.

**Conclusions:**

Our research demonstrates that iron overload occurs during VC, and that inhibition of Slc39a14 and Slc39a8 significantly relieves VC by intercepting iron overload-induced ferroptosis in VSMCs, providing new insights into the VC treatment.

**Graphical Abstract:**

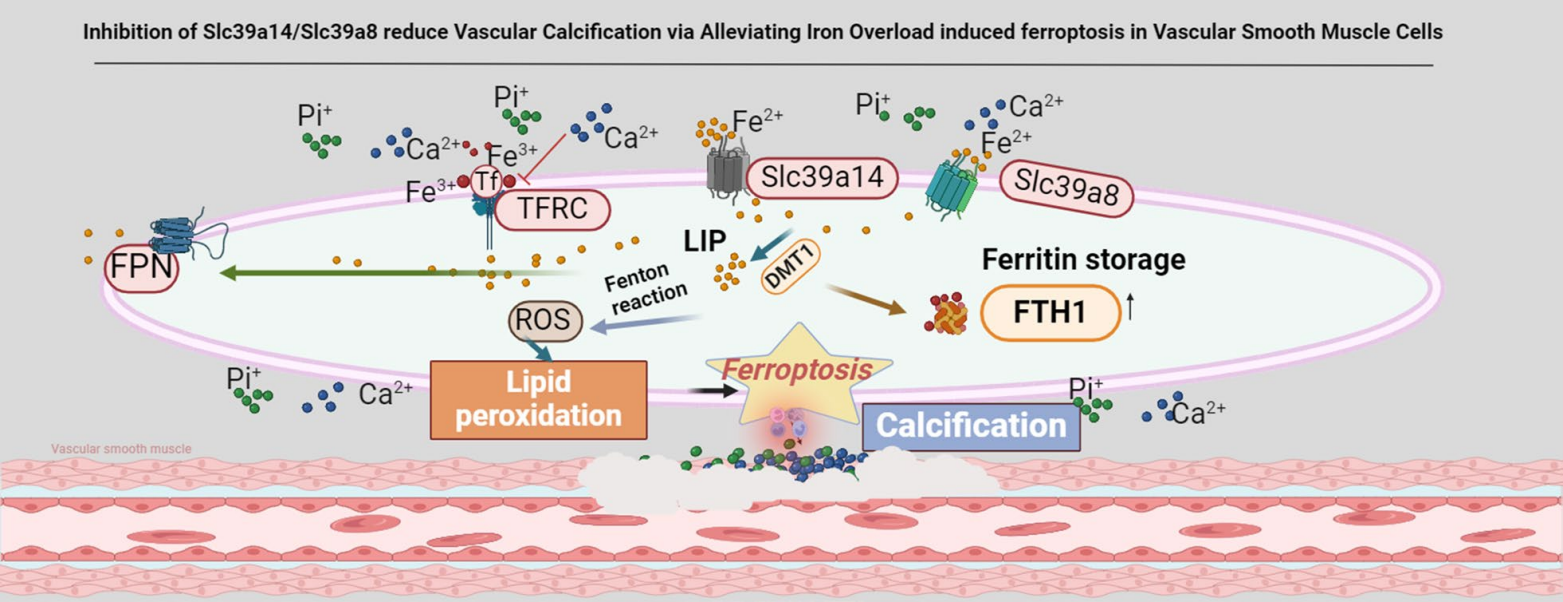

**Supplementary Information:**

The online version contains supplementary material available at 10.1186/s12933-024-02224-z.

## Background

Vascular calcification (VC) refers to the abnormal deposition of calcium phosphate crystals within the arterial walls, leading to the hardening and stiffening of blood vessels [1, 2]. It is a complex pathological process associated with a range of cardiovascular conditions including diabetes, atherosclerosis, chronic kidney failure, arterial stiffness, and cardiovascular events [3–5]. Accumulating evidence indicates that VC is an active process associated with disruption of normal vascular homeostasis and an imbalance in several regulatory factors. Various signalling pathways, including those involving molecules such as osteopontin, osteocalcin, and matrix Gla protein, play intricate roles in the onset and progression of VC [6, 7]. Oxidative stress, inflammation, and a genetic predisposition contribute to the development of this pathological process [8]. However, the precise mechanisms underlying VC remain poorly understood, and no clinical therapies are currently available to address the condition.

Recently, ferroptosis has been recognised as a novel therapeutic target for cardiovascular diseases and has been widely studied [9, 10]. It is characterised by severe lipid peroxidation, which depends on iron overload [11, 12]. Iron overload is characterised by the excessive accumulation of intracellular iron. Normally, cellular iron levels are regulated by complex iron-related signalling pathways and hepcidin. However, Disruption of the balance between iron absorption and exclusion can lead to abnormal intracellular iron levels and trigger cellular functional disorders [13–16]. As a key catalyst, free iron participates in the Fenton reaction and induces ROS production. Excessive ROS can cause mitochondrial dysfunction, oxidative stress, lipid peroxidation, and DNA damage, ultimately leading to iron-dependent cell death (ferroptosis) [17], promoting the migration and proliferation of smooth muscle cells, extracellular matrix remodelling, and osteogenic differentiation, further exacerbating the pathogenesis of VC [18, 19]. Given the role of iron in ROS formation, iron overload may exacerbate the development and progression of VC [20]. Despite recent discoveries highlighting the association between ferroptosis and VC [21], especially through the SLC7A11/GPX4/GSH signalling pathway [22, 23], the underlying regulatory mechanism of ferroptosis in VSMCs, especially with regard to iron metabolism, remains poorly understood. Related research has reported that the metalloproteins Slc39a14 and Slc39a8 are associated with iron overload in some diseases, but their functions, especially in the context of VC, are still unclear. Therefore, our aim was to explore the role of iron overload in the development of VC and the mechanisms of iron overload occurrence and to provide evidence to prevent the progression of VC through strategies that reduce the body's iron content or inhibit iron death in vascular smooth muscle cells.

## Methods

### Animal studies

C57BL/6J mice were purchased from Hua Fu Kang Bio Tech (Beijing, China) at 6–8 weeks of age. The mice were housed at five individuals per cage and used at a weight of approximately (20.0–22.0 g). All mice were housed in standard laboratory conditions with a 12-h light/12-h dark cycle and had free access to tap water and food. All mice were randomly divided into four groups (n = 6 per group), namely the control, vitamin D, DFO, and dextran groups. A mouse VC model was established by intraperitoneal injection of vitamin D3 (5 × 10^5^ IU/kg) for 3 consecutive days as previously described[24]. Mice in the DFO group received intraperitoneal injections of DFO (100 mg/kg) for 4 consecutive days. The iron-overload group with intraperitoneal injection of Iron Dextran (FeDex, 0.7 g/kg) for 4 d. After 4 weeks, the mice were anaesthetised and euthanised to collect blood and aorta samples.

### Cell culture

Primary mouse aortic smooth muscle cells (MAoSMCs) were isolated from 6-week-old C57BL/6 mice as described previously [24]. MAoSMCs were grown to confluence in Dulbecco’s modified Eagle medium:F12 medium supplemented with 10% foetal bovine serum and 1% penicillin/streptomycin(GM) (Beyotime Institute of Biotechnology, Shanghai, China) at 37 °C in 5% CO_2_ infusion and humidified air. Cells from passages 3 to 8 were used in all experiments. The medium was replaced every 2 d. To induce calcification, MAoSMCs were maintained in the presence of calcifying medium (CM) containing 10 mM β-glycerophosphate and 3 mM calcium chloride for 7 d, after which alkaline phosphatase (ALP) activity was assessed and western blotting was performed. The cells were treated for 12–14 d for calcium concentration and alizarin red staining.

### Western blotting

Western blotting analyses were performed using specific antibodies against β-actin (Affinity, AF7018), BMP2(Abcam, ab284387), RUNX2 (CST, D1L7F), FTH1 (Abclonal, A19544), FTL (Abclonal, A18051), α-SMA (Proteintech, 67735-1-lg), Slc39a8 (Proteintech, 20459-1-AP), Slc39a14 (Affinity,DF14224) and TFRC (Proteintech, 66180-1-Ig). Total protein samples were extracted from MAoSMCs and mouse aortic tissue according to a previously published method. Briefly, the samples were lysed on ice for 30 min in a mixture of radioimmunoprecipitation assay lysis buffer (EpiZyme, Cambridge, MA, USA; PC101) and protease inhibitor (p6730).The homogenates were then centrifuged at 12,000×*g* for 15 min at 4 °C. The total protein concentrations were determined using a BCA Protein Assay kit (Beyotime Institute of Biotechnology, P0012). The protein samples were mixed with 5× sodium dodecyl sulphate–polyacrylamide gel electrophoresis (SDS-PAGE) loading buffer and heated at 98 °C for 10 min. The samples were subjected to SDS-PAGE at different polyacrylamide concentrations and then transferred to polyvinylidene fluoride membranes (Millipore, Boston, MA, USA). The membranes were washed three times with TBST and incubated with specific antibodies overnight. They were then washed three times with TBST and incubated with the secondary antibody at room temperature for 1 h. Protein bands were visualised using Omni-ECL (SQ201) and images were captured using an enhanced chemiluminescence system (ChemiScope, Shanghai, China). Western blots were quantified using ImageJ analysis software (NIH, Bethesda, MD, USA) and the protein levels were normalised to β-actin levels.

### Quantitative real-time polymerase chain reaction

The relative mRNA levels of these genes were determined using real-time quantitative PCR. Briefly, total RNA was isolated from VSMCs and aortic tissue using a Total RNA Isolation Kit (RE-03111) according to the manufacturer’s protocol. RNA purity was determined based on a ratio of the absorbance at 260 to 280 nm of 1.9–2.1. cDNA was synthesised using RT Easy™ II (FORE GENE; RT-01022). Quantitative reverse transcription‒polymerase chain reaction (PCR) was performed using Real Time PCR Easy™-SYBR Green I (FORE GENE, QP-01012). The relative mRNA expression level was calculated using the delta-delta-CT method, with β-actin as the reference gene.

### Immunofluorescence staining

Cells were seeded onto slides in a 12-well plate treated with or without CM medium for 7 d then washed three times with phosphate-buffered saline (PBS) to remove the culture medium. Subsequently, the cells were fixed with 4% paraformaldehyde for 15 min and permeabilised with 0.5% Triton X-100 for 15 min. After blocking with 5% goat serum in PBS containing 0.1% Triton, the cells were washed three times with PBS and incubated overnight with the primary antibody. Following this step, they underwent three additional washes with PBS and were finally incubated at room temperature for 1–2 h with a secondary antibody. The nuclei were stained with 4′,6-diamidino-2-phenylindole (DAPI), and immunostaining was observed using OLmPYA. Tissue sections were cut at a thickness of 5 μm, briefly immersed in PBS for 3 min was to remove the OCT compound and were then stained following the same steps as those applied to the cells.

### Perls’ Prussian blue staining

Iron accumulation in tissue samples was detected using Perls’ Prussian blue staining according to the instructions provided by the manufacturer. Tissue sections were cut at a thickness of 4 μm and washed with distilled water three times. After dewaxing, the sections were incubated in Perls’ solution (5% potassium ferrocyanide [Yeasen Biotech, Shanghai, Chia]/5% hydrochloric acid) for 40 min, followed by three washes in distilled water. Nuclear Fast Red was used to stain the nuclei for 5 min, after which, the sections were washed three times with distilled water. After staining, the slices were dehydrated in a gradient ethanol series, cleared with xylene, and sealed with resinene. Images were captured using a digital pathology slide scanner (KF-PRO-002).

### Immunohistochemical staining

According to previously described methods, aortic tissues were fixed in 10% neutral formaldehyde for 24–48 h and sequentially dehydrated, embedded, sliced, and dewaxed. Antigen retrieval was performed by boiling in citrate for 20 min, and hydrogen peroxide incubation was performed at room temperature in a dark box for 10 min to inhibit endogenous peroxidases. The sections were then blocked with 0.3% Triton X-100 and 5% bovine serum albumin in PBS for 1 h. Next, the sections were incubated overnight with primary antibodies at 4 °C. The primary antibody was removed, and the sections were washed three times with PBS and then incubated with secondary antibodies (Beyotime Institute of Biotechnology) for 1 h at room temperature. Diaminobenzidine (Sigma-Aldrich, St Louis, MO, USA) was used to detect positive staining, and haematoxylin was used for counterstaining. Images were captured using a digital pathology slide scanner (KF-PRO-002) and were analysed using Image-Pro Plus software.

### Alizarin red S staining

Alizarin Red S staining was used to detect calcification, as previously described [24]. VSMCs were cultured under different conditions for 12–14 d and washed with PBS three times to remove the culture medium. They were then fixed with 4% formaldehyde for 15 min and stained with Alizarin Red S (1%, pH 4.2) for 30 min at room temperature. The cells were rinsed with distilled water to remove excess dye, and images were captured under an inverted microscope. Artery tissue sections were cut into 4 µm sections and dewaxed. The sections were stained with 1% Alizarin Red S (pH 4.2, Sigma-Aldrich) for 5 min at room temperature and then rapidly rinsed with distilled water. The sections were dehydrated, made transparent, and sealed with neutral gum. For whole-artery staining, mouse aortic tissue was fixed in 95% ethanol for 24 h, stained with 0.003% Alizarin Red in 1% potassium for another 24 h, and washed three times with 2% potassium hydroxide. Calcified areas were indicated by red staining. To quantify the extent of calcification, the stained cells were incubated with 10% formic acid for 5 min and the optical density at 405 nm was measured using a microplate reader.

### Von Kossa staining

Von Kossa staining was used to assess calcium deposition according to the manufacturer’s instructions. Artery tissue sections were cut into 4 µm sections and dewaxed. They were then incubated with 5% silver nitrate (Sigma-Aldrich) for 45 min under ultraviolet light and then washed with distilled water for 2 min, followed by incubation in sodium thiosulfate for 10 min and then rapidly rinsed with distilled water, and images were captured under an inverted microscope. After fixation in 10% formaldehyde for 24 h, the entire artery was incubated with 5% silver nitrate (Sigma-Aldrich) for 45 min under ultraviolet light and then in sodium thiosulfate for 10 min.

### Malondialdehyde assay

To analyse lipid peroxidation after treatment with calcifying medium for 7–10 d, the malondialdehyde (MDA) concentration in MAoSMCs was determined using an MDA assay kit (Beyotime Institute of Biotechnology). In brief, vascular smooth muscle sample was collected and thawed on ice for 30 min. Lysates were then centrifuged at 10,000–12,000 g at 4 °C for 10 min and the resulting supernatant was harvested. All samples were cooled on ice After incubating the reaction system in metal bath for 20 min. The samples were centrifuged at 1000×*g* for 10 min, and the MDA content was determined by measuring the absorption at 532 nm. The final concentration of MDA was calculated relative to the total amount of protein in each sample.

### Cell transfection

For knockdown, small interfering RNAs (siRNAs) targeting *SLC39A8* (siRNA-SLC39A8) and *SLC39A14* (siRNA-SLC39A14) were purchased from Ribo. siRNA-SLC39A8 (50 nM) and siRNA-SLC39A14 (50 nM) were transfected using Lipofectamine 3000 according to the manufacturer’s instructions. Briefly, cells were seeded at 70–80% confluence at transfection and cultured in Opti-MEM (Gibco, Waltham, MA, USA; #31985-070) for 6–8 h after adding the siRNA-lipid complex and for another 2–4 d in F12 medium with 10% FBS. The knockdown efficiency was confirmed by quantitative (q)PCR at 48 h after siRNA transfection.

For overexpression, a pcDNA-Flag vector encoding the mouse Slc39a14 gene was purchased from Nanjing Zebrafish Biotech Co, Ltd. Cells at 70–80% confluency are transfected with the plasmid in accordance with the manufacturer’s instructions. Briefly, the Lipofectamine 3000 reagent is gently mixed with the plasmid DNA in a serum-free medium. This mixture is then incubated for 15 min to allow for the formation of Lipofectamine-DNA complexes. The subsequent steps are identical to those used for SiRNA transfection.

### Iron assay kit

Intracellular iron levels were determined using the Cell Ferrous Iron Colorimetric Assay Kit (E-BC-K881-M; Elabscience, Houston, TX, USA) and the fluorescent indicator Phen Green SK, according to manufacturer’s instructions. Briefly, approximately 1 × 10^6^ cells were collected and homogenised on ice for 20 min with 150 μL of lysis buffer. The supernatant was collected after centrifugation at 15,000×*g* for 10 min. Then, 80 μL of the supernatant was mixed with the regent 3 and incubate at 37 °C for 10 min. The absorbance of each well was measured at 593 nm.

The cells were washed three times with PBS and incubated with Phen Green SK (5 nM) for 30 min at 37 °C. The Phen Green SK fluorescent probe was then removed and the cells were incubated in PBS for another 10 min at 37 °C. Spots were collected using an influenza microscope.

### Statistical analysis

All statistical analyses were carried out using GraphPad Prism version 8.4.0 (GraphPad Software, CA). All data were expressed as Mean ± SEM. Statistical significance was assessed by unpaired two tailed Student t-tests for comparing Mean values between 2 groups, and differences among more than 2 groups were compared using one-way ANOVA analysis of variance (ANOVA) followed by Tukey’s post hoc test (more than two groups). Statistical significance was indicated as follows: no significance (n.s.), *p < 0.05; **p < 0.01. Each experiment was repeated independently at least three times.

## Results

**Fig. 1:**
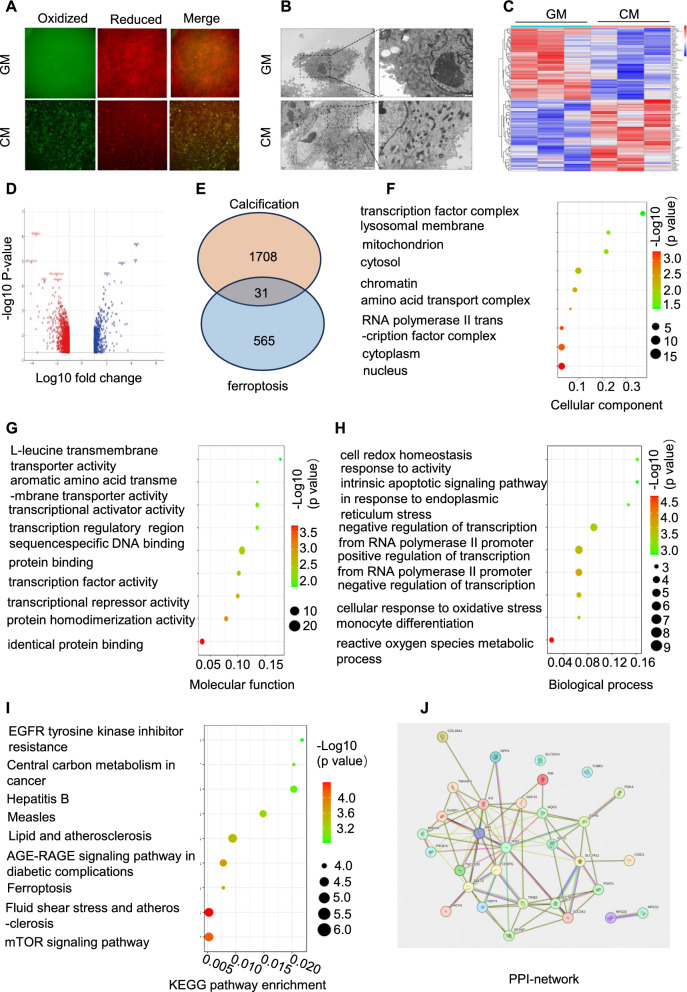
Ferroptosis contributes to vascular calcification dynamics. VSMCs were treated with high phosphate and calcium levels to induce VSMC calcification in vitro, and C11-BODIPY 581/591 was used to analyse fluorescent cytosolic and lipid ROS (**A**). Ultrastructural changes in muscle cells were examined using electron microscopy (**B**). Heatmap illustrating Differentially Expressed Genes (DEGs) between control and calcification groups in the GSE 211722 dataset (**C**). Volcano plots showing DEGs (**D**). Venn diagram illustrating overlapping DEGs identified in the two datasets (**E**). Gene Ontology enrichment analysis (biological processes, molecular functions, and cellular components) was conducted for the hub genes (**F-H**). Pathway analysis based on the Kyoto Encyclopaedia of Genes and Genomes was performed for hub genes (**I**). A Protein–Protein Interaction (PPI) network analysis was conducted for hub genes (**J**)

First, a fluorescent probe was used to examine intracellular lipid peroxides. The results showed a notable increase in the fluorescence signal due to lipid peroxides in the calcification group compared to that in the control group (Fig. 1A). Subsequently, we used electron microscopy to investigate the alterations in the intracellular organelles of VSMCs induced by high calcium and phosphate levels. Characteristic changes associated with ferroptosis such as mitochondrial shrinkage and cell membrane collapse were observed in the calcified group (Fig. 1B). Using rigorous criteria and R language analytics, 1708 differentially expressed genes (DEGs) were identified between the control and calcified groups in the GSE211722 dataset. The subsequent heat map and volcano plot representations showed a stark contrast in these DEGs between the two groups (Figs. 1C and D). By integrating data from FerrDb (http://www.zhounan.org/ferrdb/current/), an additional 565 genes were enriched, leading to the identification of 33 pivotal hub genes that are likely to be instrumental in the calcification process through the modulation of ferroptosis-related pathways (Fig. 1E). Gene Ontology (GO) functional annotation terms were systematically categorised into biological processes, molecular functions, and cellular components. Each category showed significant enrichment of the identified DEGs, particularly in the pathways associated with oxidative stress responses (Fig. 1F–I). Further analytical depth was achieved by employing the STRING database to construct a protein–protein interaction network for the 31 hub genes, promising to unlock novel insights into the ferroptosis machinery involved in VC (Fig. 1J).

**Fig. 2:**
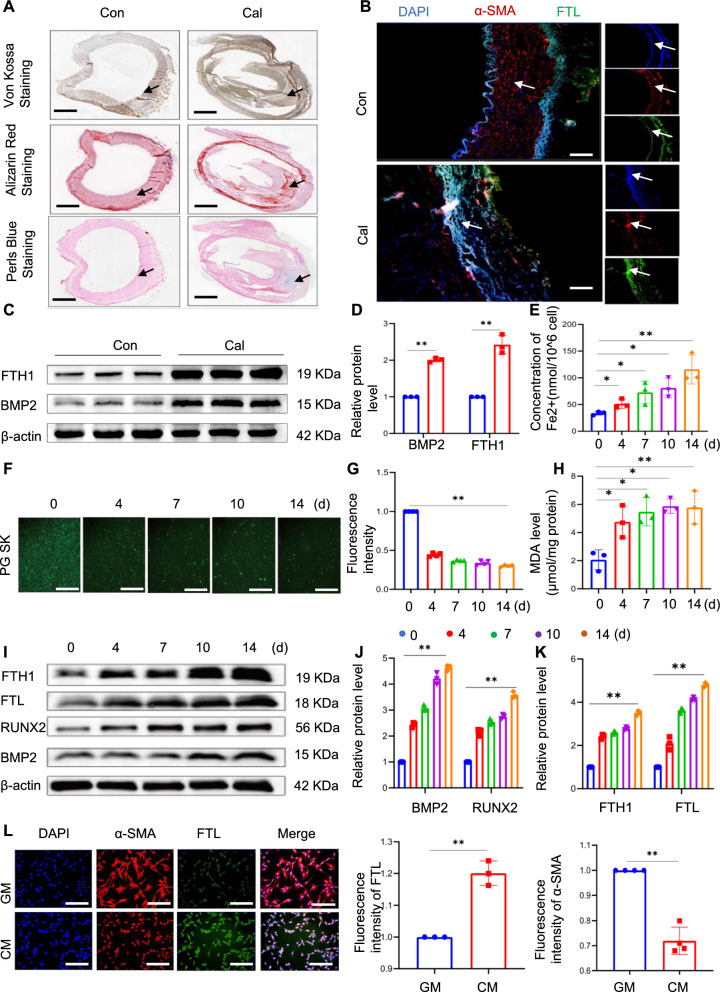
Iron overload is induced during calcium/phosphate-induced vascular calcification. Arterial specimens were obtained from the amputated patients. Artery tissue sections were cut into 5 µm thick sections. Calcification was initially confirmed through alizarin red staining and Von Kossa staining, while Perl's blue staining was employed to detect iron within the calcification area; black arrow head indicates the medial layer of artery. Scale bar: 1.25 mm (**A**). The expression of smooth muscle actin (SMA) and ferritin light chain (FTL) in human arterial samples was assessed using immunofluorescence; white arrow head indicates the medial layer of artery. Scale bar: 20 µm (**B**). Total aortic proteins were extracted and the expression of BMP2 and FTH1 was determined by western blot analysis. *P < 0.05, **P < 0.01 (**C** and **D**). MAoSMCs were treated with calcifying medium (CM) for 0, 4, 7, 10, and 14 days. The intracellular iron levels (nmol/ 10^6^ cells) were measured using an iron assay kit (E). Phen Green SK was used to evaluate intracellular Fe.^2+^. Scale bar: 50 µm (**F**). Malondialdehyde (MDA) levels were assessed (**H**). Western blot analysis of osteogenic marker proteins (Runx2 and BMP2) and iron storage proteins (FTH1 and FTL) is presented in (**I**‒**K**). The expression levels of α-SMA and FTL in VSMCs of indicated groups were determined by immunofluorescence staining (n = 4). Scale bar: 50 µm (**L**)

Ferroptosis, a form of iron-dependent cell death, culminates in the formation of lipid peroxides catalysed by divalent iron. We hypothesised that iron overload-induced ferroptosis also plays a role in the calcification of VSMCs. Calcified and normal vascular samples were obtained from the biobank of the Affiliated Hospital of Southwest Medical University to investigate whether calcified VSMCs were in a state of iron overload. Calcification was initially confirmed by von Kossa and Alizarin Red staining (Fig. 2A). Subsequently, the results of immunofluorescence staining revealed the overexpression level of FTL and low expression level of α-SMA in the calcified group compared to the control group (Fig. 2B). Western blot analysis showed a significant increase in both the storage protein FTH1 and the osteogenic protein BMP2 in the calcified group (Fig. 2C‒E). Changes in ferritin expression levels within VSMCs were further investigated using the iron-specific fluorescent dye Phen Green SK and an iron assay kit. As expected, intracellular iron levels markedly increased over time in VSMCs (Fig. 2F and G). Additionally, the levels of MDA, a prevalent by-product of lipid ROS, increased progressively over time in VSMCs after treatment with high levels of calcium and phosphate (Fig. 2H). The crucial intracellular iron storage proteins FTL and FTH1 indirectly reflect these dynamics. Consistent with the levels of Runx2 and BMP2, a significant upregulation was observed in both FTH1 and FTL protein expression over time in VSMCs, as demonstrated by western blotting (Fig. 2I–K). Immunofluorescence result also showed a significant increase in the expression level of FTL and reduction of α-SMA in the calcified VSMCs (Fig. 2L). Overall, these findings highlight the pivotal role of iron in VC and provide insights into its underlying mechanisms.

**Fig. 3:**
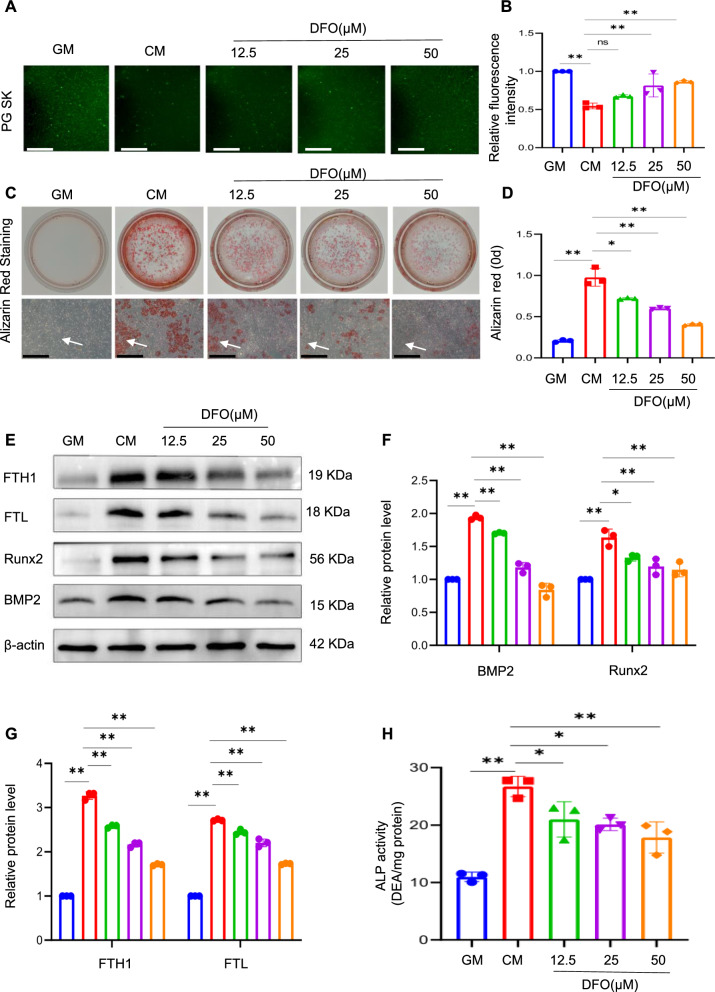
Inhibition of iron accumulation attenuates calcium/phosphate-induced VSMC calcification. Phen Green SK was used to evaluate intracellular Fe.^2+^ in VSMCs. Scale bar: 50 µm (**A** and **B**). After being treated for 14 days with GM or CM, with or without different concentrations of DFO (12.5, 25, 50 µM), calcification in VSMCs was detected by alizarin red staining and quantitative analysis. White arrow head indicates the calcified deposits. Scale bar: 100 µm (**C** and **D**). protein expression of osteogenic markers Runx2, BMP2, FTL, and FTH1 in VSMCs treated with GM or CM, with or without different concentrations of DFO (12.5, 25, 50 µM), was analysed by western blot and quantified by densitometry *P < 0.05, **P < 0.01 (**E**‒**G**). ALP activity in VSMCs was measured; *P < 0.05, **P < 0.01 (**H**)

DFO, which acts as an iron chelator, binds free iron ions and effectively reverses intracellular iron overload [32]. To gain further insights into the potential role of intracellular iron in calcification, the effect of DFO on osteogenic differentiation was investigated. VSMCs were treated with different concentrations of DFO (12.5, 25, and 50 μM) under conditions of high calcium and phosphate levels. The impact of DFO intervention on VSMC calcification was first assessed by evaluating iron levels. The fluorescence intensity within the cells increased in a concentration-dependent manner following DFO treatment, indicating that DFO effectively inhibited iron accumulation in calcified VSMCs (Fig. 3A and B). Alizarin red staining, a widely used to specifically identify calcium deposits, showed that DFO inhibited the osteogenic differentiation of VSMCs in a dose-dependent manner (Fig. 3C and D), consistent with the observed decrease in calcification. FTH and FTL levels showed a dose-dependent decrease in response to DFO treatment; the upregulation of osteogenic genes such as *Runx2* and *BMP2* was blunted by DFO treatment (Fig. 3E‒G). These findings were further supported by the quantification of ALP activity (Fig. 3H). Overall, these results provide compelling evidence for the involvement of intracellular iron homeostasis in the regulation of VSMC calcification, thereby shedding light on potential therapeutic strategies for preventing or mitigating VC.

**Fig. 4:**
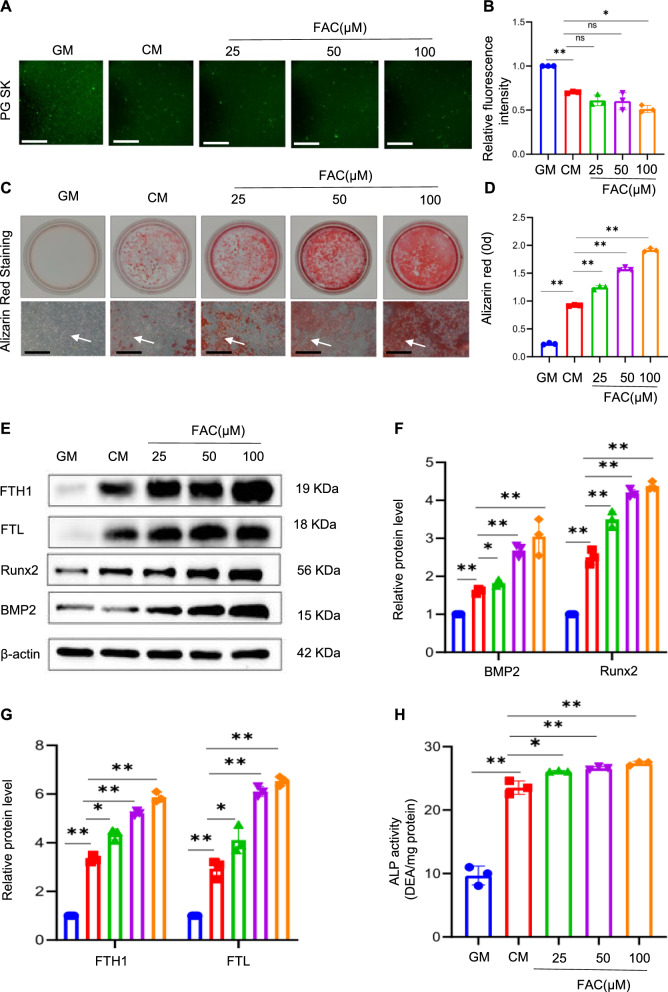
Iron overload promotes the osteogenic differentiation of VSMCs. Phen Green SK was used to evaluate intracellular Fe^2+^ in VSMCs. Scale bar: 50 µm (**A** and **B**). VSMCs were cultured in GM or CM with or without various concentrations of FAC (25, 50, 100 µM) for 14 days to detect osteogenic indicators. Alizarin red staining and quantitative analysis were conducted to determine calcium deposition. White arrow head indicates the calcified deposits. Scale bar: 100 µm (**C** and **D**). The expression of Runx2, BMP2, FTL, and FTH1 was determined by western blot and quantified by densitometry. *P < 0.05, **P < 0.01 (**E**‒**G**). ALP activity in VSMCs was measured; *P < 0.05, **P < 0.01 (**H**)

Ferric ammonium citrate (FAC) increases cellular iron levels by delivering iron ions to cells, which can be used to simulate iron overload. After treatment with FAC, it was confirmed that intracellular iron ion levels significantly increased, based on the decreased Phen Green SK fluorescence intensity (Fig. 4A and B). Several methods were employed to assess the formation of mineralised deposits, and the results clearly demonstrated that treatment with FAC led to a significant increase in calcification compared to that in the control group. Alizarin red staining intensity and quantification of the stained deposits revealed a remarkable enhancement in mineralisation in a concentration-dependent manner (Fig. 4C and D). Furthermore, the expression levels of osteogenic genes, such as *Runx2* and *BMP2* and iron storage protein genes, such as FTL and FTH1, markedly increased in a concentration-dependent manner (Fig. 4E‒G). The levels of ALP, an important marker of osteogenic differentiation, were further upregulated in the FAC group compared with those in the CM group (Fig. 4H). These results contribute to a better understanding of the effects of excess iron on osteogenic differentiation.

**Fig. 5:**
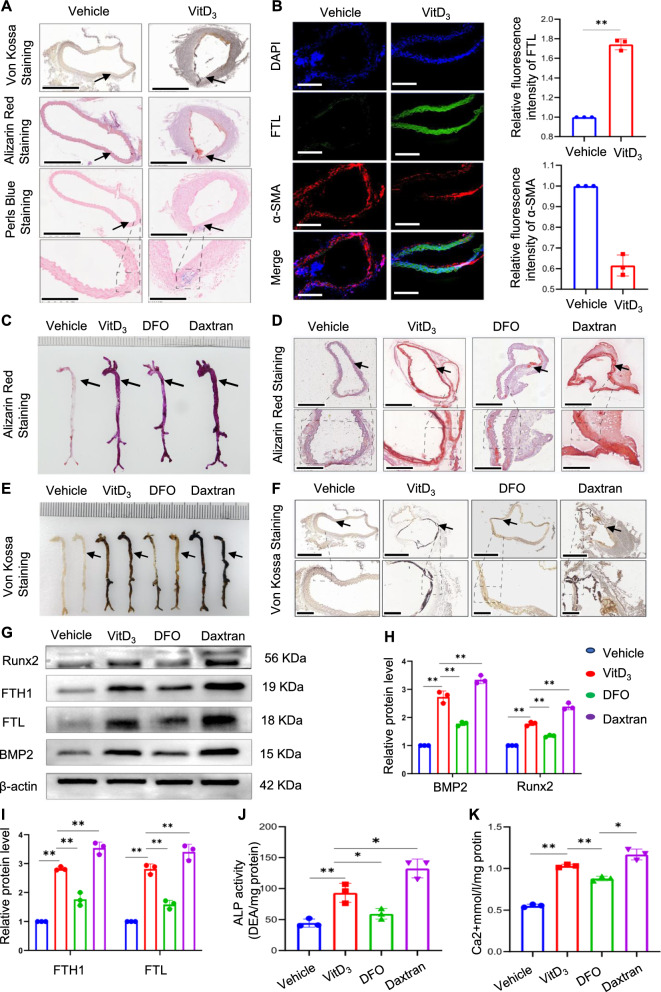
Iron overload is positively correlated with vascular calcification in mice. Mice vascular calcification was induced by injecting VitD3. Aortic sections were used to determine calcification by alizarin red and von Kossa staining; iron accumulation in aortic tissue was detected by Perl's blue staining. Black arrow head indicates the medial layer of artery. Scale bar: 300 µm (**A**). α-SMA and FTL in mouse arterial sections were assessed using immunofluorescence. Scale bar: 20 µm (**B**). The whole artery and thoracic aorta (indicated by black arrows) cross sections were used to compare calcification by using alizarin red S (**C** and **D**) and Von Kossa staining. Scale bar: 300 µm (**E** and **F**). Total aortic proteins were used to determine the expression of Runx2, BMP2, FTL, and FTH1 by western blotting (*P < 0.05, **P < 0.01, **G**–**I**). ALP activity in mouse aortas was measured (**J**). Mineral deposition in the entire artery was measured using calcium content assay (**K**)

A mouse VC model was successfully established and validated by alizarin red S and von Kossa staining (Fig. 5A). Additionally, we observed changes in iron deposition in the aorta by Perl’s Prussian blue staining, which is commonly used in medical research and pathology to detect iron deposits associated with various diseases. The results revealed a significant increase in the blue-stained area of the calcified arteries compared with that observed in the control group (Fig. 5A). Furthermore, immunofluorescence analysis of blood vessels indicated noticeable upregulation of FTL expression levels, whereas α-SMA protein expression levels were significantly decreased (Fig. 5B), suggesting a positive correlation between VC and iron deposition. Additionally, the mice were treated with two iron chelators, DFO and the iron dextran, widely used in iron-overload experiments in vivo. The effects of the iron chelators were assessed using calcification markers. Alizarin red and von Kossa staining were used to visualise and quantify the extent of aortic calcification. These results are striking. DFO treatment significantly attenuated whole aortic calcification, whereas dextran treatment exacerbated this effect (Fig. 5C‒F). The trend of protein expression levels of FTH1, FTL, Runx2, and BMP2 was consistent with that of the in vitro experiment, effectively reversing vascular calcification in mice, whereas iron overload contributed to vascular calcification (Fig. 5G‒I). The key regulators of calcification in the aorta (calcium content and ALP levels) further supported these results (Fig. 5J and K).

**Fig. 6:**
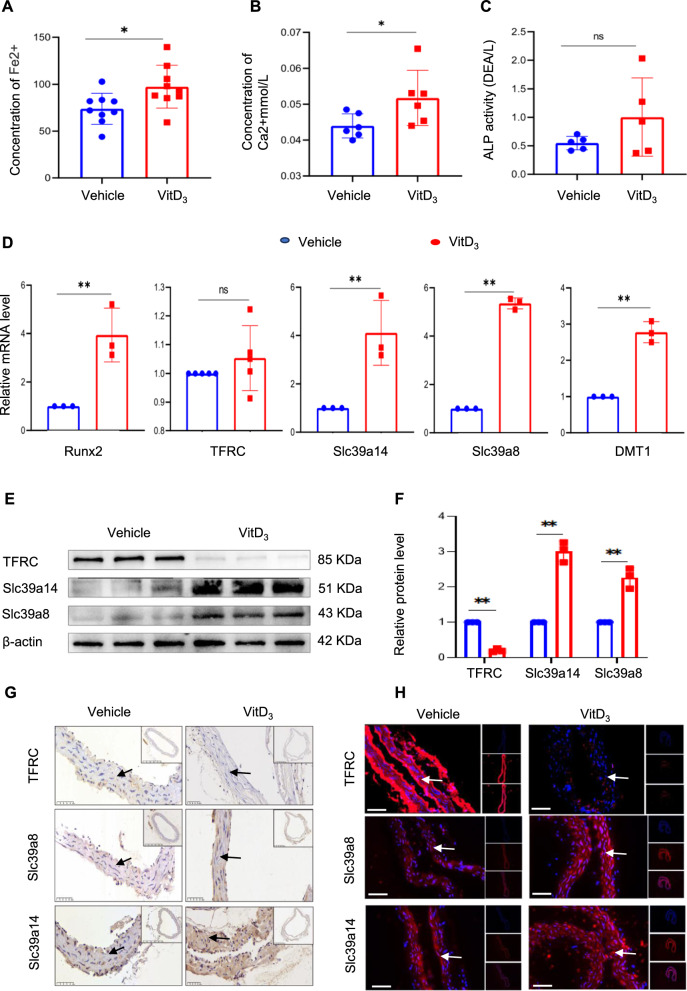
Slc39a14 and Slc39a8 are involved in the altered iron metabolism observed in vascular calcification. Venous blood was collected and serum ferrous iron levels were determined using an iron assay kit (**A**). Serum calcium and alkaline phosphatase (ALP) concentrations were measured and normalised to protein levels (**B** and **C**). Relative RNA levels of Runx2, Slc39a14, Slc39a8, DMT1, and TFRC were analysed by qPCR and normalised; *P < 0.05, **P < 0.01 (**D**). Total aortic proteins were used to determine the expression of Slc39a14, Slc39a8, and TFRC by western blotting (*P < 0.05, **P < 0.01, **E** and **F**). The expression of Slc39a14, Slc39a8, and TFRC in aortic sections was examined by immunohistochemical (IHC) staining. Black arrow head indicates the medial layer of artery. Scale bar: 50 µm (**G**). The expression levels of Slc39a14, Slc39a8, and TFRC were determined through immunofluorescence staining. White arrow head indicates the medial layer of artery. Scale bar: 20 µm (**H**)

To elucidate the mechanisms underlying the elevated iron levels in vascular tissues, we quantified serum ferrous ion concentrations in mice. The results revealed that mice with vitamin D-induced VC exhibited significantly higher divalent iron levels than the controls (Fig. 6A), along with elevated serum calcium levels without a corresponding increase in ALP levels (Fig. 6B and C). These patterns suggest a conceivable interplay between systemic iron modulation and metabolic disturbances in the organs that are integral to iron metabolism (Additional file 3). This led us to hypothesise that increased extracellular iron levels influence the expression of membrane transporters implicated in iron overload. Contrary to our expectations, there were no changes in the mRNA expression of TFR, which typically mediates iron uptake. Interestingly, the significant upregulation of Slc39a8 and SLC39A14, which are known for zinc and manganese transportation, respectively, was responsible for the cellular transport of divalent iron ions in the vitamin D-induced group (Fig. 6D). Further investigation of the expression of transporter proteins associated with the vasculature revealed a marked upregulation of SLC39a8 and SLC39A14 expression and diminished expression of TFR. Western blotting, immunohistochemical staining, and immunofluorescence staining confirmed these findings (Additional file 4) (Figs. 6E‒H).

**Fig. 7:**
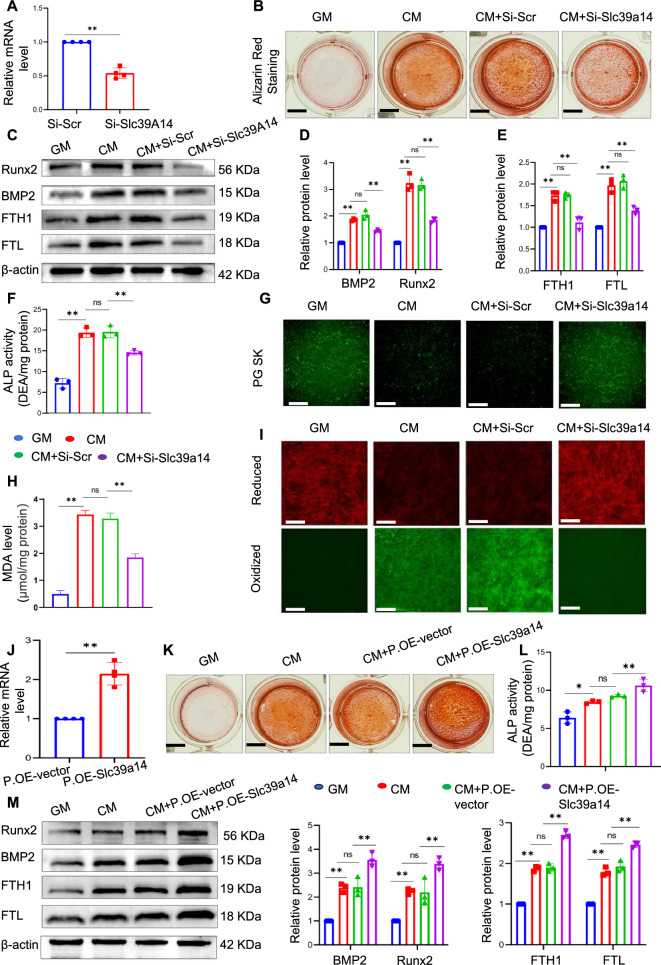
Inhibition of Slc39a14 alleviates osteogenic differentiation in VSMCs. VSMCs were transfected with siRNA against mouse Slc39a14 for 48 h, and knockdown efficiency was assessed by qPCR (**A**). Alizarin red staining was performed to evaluate calcium deposition (**B**). Total cellular proteins were analysed by western blot to determine the expression of Runx2, BMP2, FTL, and FTH1; *P < 0.05 and **P < 0.01 (**C**‒**E**). ALP activity in VSMCs was measured; *P < 0.05, **P < 0.01 (**F**). Phen Green SK was utilised to assess intracellular Fe^2+^ levels in VSMCs. Scale bar: 50 µm (**G**). Malondialdehyde (MDA) levels were measured (**H**). C11-BODIPY 581/591 was employed to analyse fluorescent cytosolic and lipid ROS. Scale bar: 50 µm (**I**). Plasmid transfection was performed to overexpress Slc39a14 in VSMCs for 48 h and the results were confirmed by qPCR (**J**). Alizarin red staining was performed to determine calcium deposition (**K**). ALP activity in VSMCs was measured; *P < 0.05, **P < 0.01, (**L**). Total cellular proteins were analysed by western blotting to determine the expression levels of Runx2, BMP2, FTL, and FTH1. Significance levels are denoted as *P < 0.05 and **P < 0.01 (**M**)

To delve deeper into the mechanisms underlying iron accumulation within VSMCs during calcification, we employed siRNA-mediated knockdown of Slc39a14 and assessed the resulting changes in internal iron levels and calcification indices. We first validated the efficiency of the knockdown by qPCR analysis (Fig. 7A). Alizarin red staining was performed to visualise the calcium deposition. Notably, we observed a marked reduction in the staining intensity in SLC39A14-silenced VSMCs compared to that in the CM group (Fig. 7B). Analysis of the protein levels of FTH1, FTL, Runx2, and BMP2 showed that knockdown of Slc39a14 reduced osteogenic differentiation and iron levels in VSMCs (C‒E). The quantification of ALP activity confirmed these results (Fig. 7F). We measured the levels of free iron within the cells and observed a noticeable decrease in intracellular iron levels in Slc39a14-silenced VSMCs compared with those in the CM group (Fig. 7G). This decrease in iron levels was consistent with the reduced expression of two key iron storage proteins, FTH1 and FTL. Our results also showed a significant reduction in MDA and ROS production in SLC39A14-silenced VSMCs, suggesting a decrease in lipid peroxidation (Fig. 7H and I). To validate our findings, we employed plasmid-mediated overexpression of SLC39A14 and confirmed its efficiency using PCR (Fig. 7J). Furthermore, we extensively investigated the alterations in calcium deposition markers and iron levels. In contrast to the results observed with gene silencing, the upregulation of SLC39A14 significantly facilitated the osteogenic differentiation of muscle cells, as evidenced by intensified alizarin red staining (Fig. 7K), augmented ALP activity ((Fig. 7L), and elevated levels of Runx2 and BMP2 proteins (Fig. 7K‒M). Increased ferritin levels indicated a further increase in cellular iron content compared to the control group following Slc39a14 overexpression (Fig. 7M). This suggests that Slc39a14 plays a significant role in iron accumulation in the VSMCs.

**Fig. 8:**
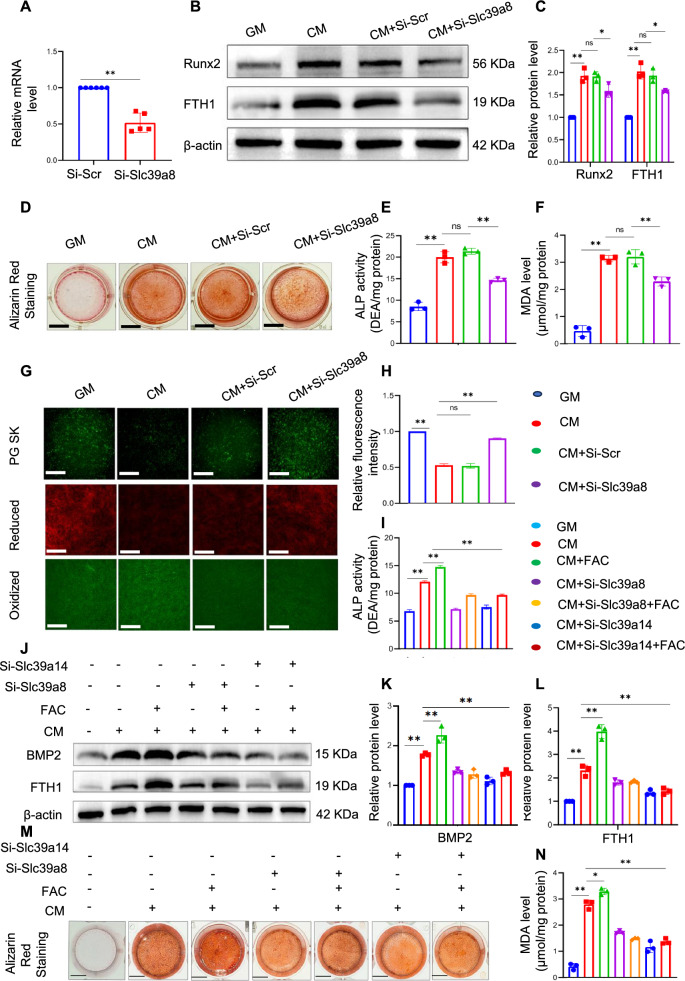
Slc39a8 participates in the regulation of iron overload during the calcification of VSMCs. VSMCs were transfected with siRNA against mouse Slc39a8 for eight hours and then switched to complete DMEM/F12 medium; knockdown efficiency was assessed by qPCR 48 h after transfection (**A**). Total cellular proteins were used to determine the expression of Runx2 and FTH1 by western blotting; *P < 0.05 and **P < 0.01 (**B** and **C**). Alizarin red staining was performed to assess calcium deposition (**D**). ALP activity in VSMCs was measured; *P < 0.05, **P < 0.01 (**E**). The levels of malondialdehyde (MDA) were also assessed (**F**). Phen Green SK was used to evaluate intracellular Fe^2+^. C11-BODIPY 581/591 was employed to analyse the fluorescent cytosolic and lipid ROS in VSMCs. Scale bar: 50 µm (**G** and **H**). Following this, the cells were switched to calcification medium (CM) or CM with or without ferric ammonium citrate (FAC), and ALP activity in VSMCs was measured again; *P < 0.05, and **P < 0.01 (I). Total cellular protein was used to determine the expression of Runx2 and FTH1 by western blotting. *P < 0.05, and **P < 0.01 (**J**‒**L**). Alizarin red staining was performed to determine calcium deposition (**M**). The levels of malondialdehyde (MDA) were assessed (**N**)

We observed high expression of Slc39a8 in the CM group. However, the role of Slc39a8 in iron overloading and calcification remains unclear. To further investigate the underlying mechanism, siRNA was used to knock down Slc39a8 and the efficiency of silencing was confirmed by qPCR analysis (Fig. 8A). Similar to the silencing of Slc39a14, silencing of slc39a8 resulted in a significant reduction in protein expression level of FTH1 and Runx2 (Fig. 8B and C). Slc39a8 knockdown relieved osteogenic differentiation in VSMCs, including alleviation of calcium deposition and reduced ALP activity (Fig. 8D and E). Our results showed a significant reduction in MDA level in the SiRNA-slc39a8 group compare to the CM group (Fig. 8F). Inhibition of Slc39a8 also reversed intracellular iron overload and mitigated lipid peroxidation (Fig. 8G and H). Moreover, upon further investigation, we introduced FAC based on the knockdown results. Surprisingly, both Slc39a8 and Slc39a14 reduced the absorption of FAC and inhibited osteogenic differentiation of VSMCs. Reduced ALP activity (Fig. 8I), decreased levels of the osteogenic-related protein BMP2 (Fig. 8J and K), and Alizarin red staining (Fig. 8M) further support this observation. A significant reduction in MDA levels was observed in the Slc39a8 and Slc39a14 silencing groups compared to the FAC group. These results suggest a potential role for Slc39a8 and Slc39a14 in regulating iron absorption and inhibiting osteogenic differentiation of VSMCs.

## Discussion

VC manifests as a gradual deposition of calcium in the arterial walls, resulting in diminished arterial elasticity and the development of luminal narrowing [1, 25]. Numerous studies have indicated that VC is an active process that disrupts the normal vascular homeostasis and involves an imbalance in several regulatory factors. Oxidative stress, inflammation, and genetic predisposition contribute to the development of this pathological process [26, 27].

Ferroptosis, a non-apoptotic form of cell death, is iron-dependent and is characterised by lipid peroxidation. Disruption of iron homeostasis results in iron overload, where excess ferrous iron (Fe^2+^) induces lipid peroxidation via the Fenton reaction, leading to a redox imbalance [28–30]. A growing body of evidence underscores the pivotal role of ferroptosis in the pathogenesis of numerous cardiovascular diseases by disrupting normal cellular functions [31–33]. For instance, oxidized low-density lipoprotein (ox-LDL) has been implicated in the induction of endothelial dysfunction and ferroptosis through the modulation of ADM transcription and the activation of the AMPK signaling cascade [34]. Furthermore, Fang et al. have elucidated that ferroptosis is instrumental in the pathogenesis of DOX-induced cardiomyopathy [35]. Recent studies have predominantly focused on the classical GPX4-GSH signalling pathway because of its significant antioxidant function in eukaryotes. Recent research has suggested a close association between the GPX4-GSH system, arterial atherosclerosis, and vascular calcification, potentially linked to the phenotypic transformation of VSMCs and Fer-1,ferroptosis inhibitor, is effective in mitigating vascular calcification, while ferroptosis-inducing drug erastin can significantly accelerate calcification in the aortic ring [21, 22, 36, 37]. Iron is an essential element for ferroptosis and plays an important role in lipid peroxide formation. However, compared with the classical pathway, iron metabolism has been less studied in diseases. A previous study showed that iron overload promotes atherosclerosis [38]. Compared with normoferremic ApoE−/− mice, atherosclerosis is profoundly aggravated in iron-loaded ApoE−/− FPNwt/C326S mice, demonstrating that non-transferrin-bound iron (NTBI)-triggered iron overload aggravates atherosclerosis and reveals a causal link between NTBI and the progression of atherosclerotic lesions. Although VC and atherosclerosis share many pathological foundations, as both entail the phenotypic transition of vascular smooth muscle cells [8], their mechanisms of occurrence differ significantly, and the mechanism by which iron overload contributes to VC remains unclear.

Studies have demonstrated that the maintenance of systemic iron homeostasis relies on a delicate balance between iron uptake, recycling, and loss [39]. Disruption of this equilibrium within cells can lead to the abnormal accumulation or depletion of intracellular iron ions [40, 41]. Under normal circumstances, the regulation of iron homeostasis in the cardiovascular system is mediated by the binding of transferrin (containing two trivalent iron molecules) to its receptor, TFRC, triggering clathrin-dependent endocytosis of the entire complex into the cells. Subsequently, vesicular ATPases acidify the endosome, while the six-transmembrane epithelial antigen prostate family of metalloreductases reduces trivalent to divalent iron [42]. The released divalent iron enters the cytoplasm via NRAMP2 (also known as DMT1), whereas cytoplasmic ferroportin oxidises ferrous ions to ferric ions, allowing ferritin-bound iron to be either degraded for enzymatic reactions or stored via ferritin (FTH1 and FTL) [43]. Iron-saturated ferritin undergoes degradation through autophagy mediated by NCOA4, a process referred to as ferritinophagy, resulting in intralysosomal ferritin degradation and release of iron, which is transported into the cytoplasm via lysosomal NRAMP2. This mechanism contributes to iron overload [44]. Another portion of the iron is transported out of the cytoplasm through the facilitated transport protein FPN, which is the only known iron exporter in vertebrate cells [45]. Cardiac iron overload, impaired heart function, and shortened lifespan have been observed in mice with early cardiomyocyte-specific deletion of the *Fpn* gene.

Slc39a14 and Slc39a8 are key members of the zinc transporter protein family (SLC39 or ZIP), and are particularly important for regulating the balance of metal ions within cells, especially for stabilising trace elements such as zinc and iron [46]. Although their primary functions involve absorption and regulation of zinc, studies have suggested that they may influence iron metabolism. Notably, among all ZIP transporters, Slc39a14 stands out because of its broad role in the integration of physiological functions [47]. Initially, Slc39a14 was used to accumulate information on uncharacterized human gene sequences. This protein contains multiple transmembrane domains responsible for the identification and transport of metal ions [48].

In terms of iron regulation, studies have shown that Slc39a14 can transport non-transferrin bound iron (NTBI), and increased iron accumulation has been observed in HEK293 human kidney cells and Sf9 insect cells overexpressing mouse Slc39a14 [49]. In contrast, iron accumulation was reduced in AML12 liver cells when Slc39a14 expression was inhibited. Furthermore, studies have revealed that the removal of the gene encoding Slc39a14 in mouse models significantly decreases the uptake of NTBI by the liver and pancreas [50]. By crossbreeding Slc39a14-deficient mice with mouse models of haemochromatosis (Hfe and Hfe2 mutants), researchers explored the role of Slc39a14 in tissue iron loading. These results indicate that iron loading in the livers of haemochromatotic mice lacking Slc39a14 was significantly reduced, which also prevented iron deposition in the liver and pancreatic cells. These findings suggest that inhibiting the function of Slc39a14 may help alleviate the liver and pancreatic iron burden and the associated pathological changes in iron overload diseases. Neurodegenerative diseases and inflammation are associated with disturbances in iron homeostasis, leading to increased NTBI concentrations in the brain. Studies have shown that glial cells, both in vivo and in vitro, upregulate Slc39a14 expression under inflammatory and iron-exposure conditions and that Slc39a14 is particularly important for the uptake of iron by astrocytes [51].

Comparative analysis of members of the Slc39 subfamily has shown that Slc39a14 and Slc39a8 are closely related evolutionarily [52]. Slc39a8 is highly conserved in all vertebrates and encodes a Slc39a8 metal cation transporter. Compared with Zn^2+^, Mn^2+^ has been identified as the preferred physiological substrate for Slc39a8 [53]. This has been linked to a reduction in ^54^Mn uptake in the brains of Slc39a8 iKO mice and the effective inactivation of Slc39a8 in isolated cerebral micro vessels, indicating that Slc39a8 mediates Mn uptake by the brain through the blood–brain barrier [54]. Subsequently, Fe^2+^ and Co^2+^ were reported as substrates for Slc39a8. Although Slc39a8 can also transport iron, its specific role in iron regulation during steady-state and pathological processes remains poorly understood [55]. The global knockout of Slc39a8 in mice results in early embryonic lethality.Consequently, compared to Slc39a14, there is relatively less research on the mechanism of Slc39a8 [52]. Recent studies have reported increased tubular iron deposition in patients with chronic kidney disease with elevated Slc39a8 levels [56]. Recent research has revealed that the metal ion transporter protein Slc39a8 plays a pivotal role in the ferroptosis-mediated degeneration of RPE cells. Transcriptomic analyses have shown an upregulation of Slc39a8 in patients with AMD (Age-related Macular Degeneration). This upregulation has also been observed in RPE cells under oxidative stress and in mouse models of AMD. Importantly, the degeneration of RPE cells can be reversed and retinal function can be restored by specifically blocking Slc39a8, thereby improving vision loss in a mouse model induced by NaIO_3_. Mechanistically, the accumulation of intracellular iron mediated by Slc39a8 is crucial, leading to an increase in lipid peroxidation and a consequent increase in RPE cell death [57]. Although the role of Slc39a14 and Slc39a8 in iron transport is continually being studied, it is still unclear whether they regulate the occurrence of iron overload in vascular smooth muscle cells during the calcification process. Our research has found that Slc39a14 and SLC39a8 are significantly elevated in calcified groups, and inhibition and enhancement experiments confirm that they regulate the occurrence of ferroptosis induced by iron overload in vascular smooth muscle cells. However, it is important to note that in our research, we did not observe differences between the two in their effects on iron metabolism, both of which regulate iron transport in vascular smooth muscle cells. Further studies are needed to understand how they function, their dominant roles, and how they work together [54].

Interestingly, we reported that FAC promoted VC in the VSMCs, which contradicts previous findings suggesting that iron citrate inhibits high Pi-induced Ca deposition by prevention of apoptosis, induction of autophagy, and partially affecting osteoblastic differentiation [58, 59]. This raises the question of why these differences exist. We identified several potential factors contributing to these disparities. First, there are differences in the modelling methods used. In contrast to our approach, some studies have utilised inorganic phosphorus for modelling, and relevant studies have reported that iron reacts with phosphate ions to form precipitates [58, 60]. The specific form of iron phosphate precipitation depends on the phosphate ion composition in the reaction. For instance, when phosphate exists in the form of dihydrogen phosphate ions (H_2_PO_4_^−^), it may lead to the formation of iron dihydrogen phosphate precipitates [61, 62]. Consequently, the observed mitigating effect of FAC on calcification may be attributed not to iron overload, but rather to a direct reduction in phosphate concentration in the culture medium. We compared inorganic phosphorus with organic phosphorus and detected a significant inhibitory effect of FAC on calcification in the inorganic phosphate groups, whereas in the organic phosphate group, it promoted calcification (Additional file 2). Moreover, variations in treatment duration may have contributed to these differences. The treatment duration was relatively short in our in vitro experiments [59].

In summary, our findings indicate that iron overload mediated by Slc39a14 and Slc39a8 in VSMCs promotes VC. The inhibition of intracellular iron or suppression of the transport proteins Slc39a14 and Slc39a8 may play a role in inhibiting iron-induced ferroptosis and preventing VC. From a clinical perspective, these observations underscore the need for further investigation of changes in iron levels as an early diagnostic method, and iron suppression as a potential therapeutic strategy for preventing VC. However, relying solely on serum iron testing to reflect the intracellular iron metabolism in VSMCs is challenging because of the presence of other pathological conditions in clinical samples. Therefore, whether reliable biomarkers can be used to predict changes in iron status in cardiovascular diseases remains uncertain. Currently, dexrazoxane, an FDA-approved cyclic derivative of ethylenediaminetetraacetic acid with high cell membrane permeability and the ability to bind with intracellular free iron, is the only drug available for preventing doxorubicin-induced cardiotoxicity in cancer patients. Recent studies have proposed that dexrazoxane exerts its cardioprotective effects mainly by inhibiting ferroptosis [35]; however, this raises the question of why other iron chelators are ineffective against DOX-induced heart damage. Research has suggested that the ability of dexrazoxane to directly enter the cardiac mitochondria and reduce iron accumulation sets it apart from other chelators that cannot penetrate the mitochondria effectively. This finding is supported by studies demonstrating that Mito-Ferro Green (a novel mitochondria-specific iron chelator) provides cardiac protection during doxorubicin treatment in mice. This led us to consider that the specific inhibition of cell membrane Slc39a14 and Slc39a8 may prevent intracellular iron overload from exerting protective effects. However, this hypothesis requires further investigation.

### Supplementary Information

**Additional file 1:** **S-1.** Iron overload promotes the osteogenic differentiation of VSMCs. **S-2.** Regulation of iron homeostasis is a complex process. **S-3**. High phosphate and calcium induce upregulation of Slc39a14 and Slc39a8 in VSMCs.

**Additional file 2: Figure S1.** After being treated with 5mM Inorganic phosphate, Calcification in VSMCs was detected by Alizarin Red staining (A).protein expression of osteogenic markers (BMP2), ALP and iron storage protein FTH1 was analyzed by Western blot and quantified by densitometry *P < 0.05, **P < 0.01 (B-D). ALP activity in VSMCs was measured, *P < 0.05, **P < 0.01 (E).VSMCs were treated with 10 mM β-glycerophosphate and (3 mM calcium chloride) for 7 d, and protein expression of (BMP2), ALP and FTH1 was analyzed by Western blot and quantified by densitometry *P < 0.05, **P < 0.01 (F-H).ALP activity in VSMCs was measured, *P < 0.05, **P < 0.01 (I).

**Additional file 3: Figure S2.** Perls blue staining were used for detection of iron accumulation in duodenum, liver and spleen, black arrow head indicated the iron deposition, Scale bar:100µm (A).Total tissue RNA were used to determine the expression of Slc39a14, Slc39a8, and TFRC by qPCR in the Duodenum,Spleen and Liver *P < 0.05, **P < 0.01 (B-D).

**Additional file 4: Figure S3.** Relative RNA levels of Runx2, Slc39a14 and Slc39a8 were analyzed by qPCR and normalized, *P < 0.05, **P < 0.01(A-C).The expression levels of Slc39a14 and Slc39a8 were determined through immunofluorescence staining, Scale bar:50µm (D-E).

## Data Availability

No datasets were generated or analysed during the current study.

## Abbreviations

DEG: Differentially expressed gene
DFO: Deferoxamine
GO: Gene Ontology
MDA: Malondialdehyde
qPCR: Quantitative polymerase chain reaction
ROS: Reactive oxygen species
SDS-PAGE: Sodium dodecyl sulphate–polyacrylamide gel electrophoresis
TBST: Tris-buffered saline with Tween 20
VC: Vascular calcification
VSMCs: Vascular smooth muscle cells
GM: Growth medium
CM: Calcifying medium
VitD3: Vitamin D3
RUNX2: Runt related transcription factor 2
BMP2: Bone morphogenetic protein 2
FTH1: Ferritin heavy chain 1
FTL: Ferritin light chain
α-SMA: Smooth muscle actin
TFRC: Transferrin receptor
Slc39a14: Solute carrier family 39 member 14
Slc39a8: Solute carrier family 39 member 8
DMT1: Solute carrier family 11 member 2
NTBI: Non-transferrin bound iron
