# Supplementary material for: Inhibition of Slc39a14/Slc39a8 reduce vascular calcification via alleviating iron overload induced ferroptosis in vascular smooth muscle cells
Source: Cardiovasc Diabetol. 2024 May 29;23:186. doi: 10.1186/s12933-024-02224-z (PMC11138056; doi:10.1186/s12933-024-02224-z)
Supplement: Supplementary file 1 — Additional file 1: S-1. Iron overload promotes the osteogenic differentiation of VSMCs. S-2. Regulation of iron homeostasis is a complex process. S-3. High phosphate and calcium induce upregulation of Slc39a14 and Slc39a8 in VSMCs. [file 12933_2024_2224_MOESM1_ESM.docx]

**Inhibition of** **Slc39a14/Slc39a8 reduce Vascular Calcification via Alleviating Iron Overload induced ferroptosis in Vascular Smooth Muscle Cells**

Yierpani Aierken^a,†^, Huqiang He^a,b,c,†^ , Runwen Li ^a^, Zipeng Lin ^a^, Tongjie Xu ^a^, Li Zhang ^a^ , Ya Wu^a,d,*^, Yong Liu^a,b,c,d, *^

a Department of Vascular Surgery, The Affiliated Hospital, Southwest Medical University, Luzhou, 646000, China

b Key Laboratory of Medical Electrophysiology, Ministry of Education & Medical Electrophysiological Key Laboratory of Sichuan Province, (Collaborative Innovation Center for Prevention of Cardiovascular Diseases) Institute of Cardiovascular Research, Southwest Medical University, Luzhou, 646000, China.

c Department of General Surgery, The Affiliated Hospital, Southwest Medical University, Luzhou, 646000, China

d Metabolic Vascular Disease Key Laboratory of Sichuan Province, The Affiliated Hospital, Southwest Medical University, 646000 Luzhou, China.

* Corresponding author. Department of Vascular surgery, The Affiliated Hospital, Southwest Medical University, No. 25, Taiping Street, Luzhou, 646000, Sichuan Province, China. E-mail address: lyong74@swmu.edu.cn (Yong Liu), E-mail: yawu@swmu.du.cn (Ya Wu)

†These authors contributed equally to this work.

Institutional email addresses for authors: 20210299120089@stu.swmu.edu.cn(Yierpani Aierken), hehuqiang047@swmu.edu.cn(HuQiang He),20220299120424@stu.swmu.edu.cn(Runwen Li), 20210299120415@stu.swmu.edu.cn (Zipeng Lin), xutongjie108@swmu.edu.cn (Tongjie Xu), 20210299120090@stu.swmu.edu.cn (Li Zhang)

**Additional Material**

**S-1 Iron overload promotes the osteogenic differentiation of VSMCs**

Some studies reported that ferric ammonium citrate (FAC) mitigates vascular calcification in vitro. However, in our experiments, FAC was found to promote the calcification of vascular smooth muscle cells. To uncover the reasons behind this discrepancy, we designed two different calcification models using 5 mM phosphate (inorganic phosphate) and 10 mM β-glycerophosphate and 3 mM calcium chloride to induce calcification in VSMCs. Initially, Alizarin Red staining was used to assess calcium deposition, indicating that FAC significantly inhibited calcium deposition in the inorganic phosphate group (Figure S1A). Compared to the control group (CM), the FAC-treated group exhibited a marked increase in the expression of the iron storage protein ferritin heavy chain 1 (FTH1) and the bone morphogenetic protein 2 (BMP2). However, the expression of FTH1 showed no significant difference between the GM and CM groups (Figure S1B-D). Quantitative assessment of alkaline phosphatase (ALP) activity suggested that FAC reduces its activity level (Figure S1E). Contrasting trends were observed in the organic phosphate group, where FAC promoted osteogenic differentiation in VSMCs, with significantly higher expression of the osteogenic proteins ALP and BMP2, as well as the iron storage protein, compared to the CM group (Figure S1F-H). Quantitative results of ALP further confirmed the aforementioned findings (Figure S1I).

After being treated with 5mM Inorganic phosphate, Calcification in VSMCs was detected by Alizarin Red staining (A).protein expression of osteogenic markers Runx2, ALP and iron storage protein FTH1 was analyzed by Western blot and quantified by densitometry *P < 0.05, **P < 0.01 (B-D). ALP activity in VSMCs was measured, *P < 0.05, **P < 0.01 (E).VSMCs were treated with 10 mM β-glycerophosphate and 10 mM β-glycerophosphate for 7 d,and protein expression of Runx2, ALP and FTH1 was analyzed by Western blot and quantified by densitometry *P < 0.05, **P < 0.01 (F-H).ALP activity in VSMCs was measured, *P < 0.05, **P < 0.01 (I).

**S-2 Regulation of iron homeostasis is a complex process**

In our study, we observed a significant elevation in divalent iron levels in the serum of mice injected with vitamin D3. We employed Perls' blue staining to detect alterations in iron levels, which closely reflect changes in iron metabolism, in the duodenum, liver, and spleen. Our findings revealed a pronounced increase in iron levels in the duodenum and spleen of the calcification group, while no significant alteration was detected in the liver (Figure S2A). Previous research has highlighted the intricate regulation of iron metabolism within the body, with specific attention to transport proteins such as Slc39a14 and Slc39a8, which play distinct roles in various diseases. Consequently, we analyzed the expression of these transport proteins, closely associated with iron metabolism, in the duodenum, liver, and spleen of the calcification group. The results demonstrated a marked decrease in the expression of Slc39a14, Slc39a8, and DMT1 in the duodenum, while TFRC expression remained unaltered (Figure S2B). Conversely, in the spleen, the expression of Slc39a14, Slc39a8, and DMT1 was significantly increased, with no noticeable change in TFRC levels (Figure S2C). In the liver, the expression of Slc39a14, Slc39a8, and TFRC was reduced, while DMT1 expression did not exhibit significant variation (Figure S2D).

Perls blue staining were used for detection of iron accumulation in duodenum, liver and spleen, black arrow head indicated the iron deposition, Scale bar:100µm (A).Total tissue RNA were used to determine the expression of Slc39a14, Slc39a8, and TFRC by qPCR in the Duodenum,Spleen and Liver *P < 0.05, **P < 0.01 (B-D).

To further verify the expression level of Slc39a14/Slc39a8 in VSMCs, we employed qPCR to assess their relative expression levels. The results indicated that, compared to the control group, the expression of Slc39a14/Slc39a8 was significantly increased in the calcification group (Figure S3A-C). Immunofluorescence findings further corroborated the above results (Figure S3D).

Relative RNA levels of Runx2, Slc39a14 and Slc39a8 were analyzed by qPCR and normalized, *P < 0.05, **P < 0.01(A-C).The expression levels of Slc39a14 and Slc39a8 were determined through immunofluorescence staining, Scale bar:50µm (D-E).
